# Molecular Pathological Characteristics of Thyroid Follicular-Patterned Tumors Showing Nodule-in-Nodule Appearance with Poorly Differentiated Component

**DOI:** 10.3390/cancers14153577

**Published:** 2022-07-22

**Authors:** Mayu Ueda, Katsuya Matsuda, Hirokazu Kurohama, Zhanna Mussazhanova, Yerkezhan Sailaubekova, Hisayoshi Kondo, Tomoki Shimizu, Nami Takada, Yuki Matsuoka, Chieko Otsubo, Shinya Sato, Hiroyuki Yamashita, Atsushi Kawakami, Masahiro Nakashima

**Affiliations:** 1 Department of Tumor and Diagnostic Pathology, Atomic Bomb Disease Institute, Nagasaki University Graduate School of Biomedical Sciences, Nagasaki 852-8523, Japan; mayu.u@nagasaki-u.ac.jp (M.U.); katsuya@nagasaki-u.ac.jp (K.M.); kurohama-h@nagasaki-u.ac.jp (H.K.); aa84205256@ms.nagasaki-u.ac.jp (Z.M.); bb55b21803@ms.nagasaki-u.ac.jp (Y.S.); bb55b20007@ms.nagasaki-u.ac.jp (Y.M.); otsuboc@nagasaki-u.ac.jp (C.O.); 2 Department of Endocrinology and Metabolism, Nagasaki University Graduate School of Biomedical Sciences, Nagasaki 852-8501, Japan; atsushik@nagasaki-u.ac.jp; 3 Department of Diagnostic Pathology, Nagasaki University Hospital, Nagasaki 852-8501, Japan; 4 High Medical School, Faculty of Medicine and Health Care, Al-Farabi Kazakh National University, Almaty 050040, Kazakhstan; 5 Biostatistics Section, Division of Scientific Data Registry, Atomic Bomb Disease Institute, Nagasaki University, Nagasaki 852-8523, Japan; hkondo@nagasaki-u.ac.jp; 6 Medical Student Research Program, Nagasaki University School of Medicine, Nagasaki 852-8523, Japan; bb20116049@ms.nagasaki-u.ac.jp; 7 Medical Student Research Program, Oita University School of Medicine, Oita 879-5593, Japan; m1741107@oita-u.ac.jp; 8 Yamashita Thyroid Hospital, Fukuoka 812-0034, Japan; shinya.s.48128@kojosen.com (S.S.); yamaftc@kojosen.com (H.Y.)

**Keywords:** thyroid, nodule-in-nodule, poorly differentiated component, 53BP1, NRAS, genomic instability, immunofluorescence, Ki-67, DNA damage response

## Abstract

**Simple Summary:**

Poorly differentiated thyroid carcinoma (PDTC) is aggressive and is reportedly evoked by well-differentiated thyroid follicular-patterned tumors (TFTs). TFTs showing nodule-in-nodule (NN) appearance with PD component (PDc) but neither invasion nor metastasis are uncommon and are regarded as benign nodules despite their high-grade histological features. Our study aimed to preliminarily assess the potential role of PDc in NN in PDTC carcinogenesis using dual-color immunofluorescence of 53BP1 as a DNA damage response (DDR) molecule and the Ki-67, *NRAS codon 61*, and *TERT*-promoter (*TERT*-p) mutations. The prevalence of abnormal type 53BP1 expression and *NRAS* and *TERT*-p mutations in PDc was comparable to that of carcinomas. Because co-expression of 53BP1 and Ki-67 can be an indicator of altered DDR, the development of PDc in NN may be associated with DDR impairments after harboring *NRAS* and *TERT*-p mutations. Therefore, PDc in NN is potentially a precursor lesion associated with PDTC.

**Abstract:**

Thyroid follicular-patterned tumors (TFTs) showing nodule-in-nodule (NN) appearance with poorly differentiated component (PDc) but neither invasion nor metastasis are diagnosed as benign nodules. Although PDc exhibits histologically aggressive features relative to the outer nodule (Out-N), its pathological significance remains unclear. TP53 binding protein-1 (53BP1) is a DNA damage response (DDR) molecule that rapidly localizes at DNA double-strand breaks. Using dual-color immunofluorescence with Ki-67, the profile of 53BP1 expression is shown to be significantly altered during diverse tumorigenesis. In this study, we aimed to elucidate the malignant potential of PDc at the molecular level. We analyzed the profile of 53BP1 expression and *NRAS codon 61* and *TERT*-promoter (*TERT*-p) mutations in 16 cases of TFTs showing NN with PDc compared to 30 adenomatous goiters, 31 follicular adenomas, 15 minimally invasive follicular carcinomas (FCs), and 11 widely invasive FC cases. Our results revealed that the expression level of abnormal type 53BP1 and incidence of *NRAS* and *TERT*-p mutations in PDc were comparable to FCs, suggesting a malignant potential. Because co-expression of 53BP1 and Ki-67 can be an indicator of altered DDR, the development of PDc in NN may be associated with DDR impairments after harboring *NRAS* and *TERT*-p mutations.

## 1. Introduction

The malignant potential of thyroid follicular-patterned tumors (TFTs) other than the follicular variant of papillary carcinoma is histologically evaluated by the presence of capsular or vascular invasion or distant metastasis [1]. TFTs showing the histological feature of nodule-in-nodule (NN) appearance with a poorly differentiated component (PDc), such as a solid/trabecular/insular (STI) pattern, but neither invasion nor distant metastasis are normally diagnosed as benign nodules, such as adenomatous goiter (AG) or follicular adenoma (FA). Although PDc in NN is histologically similar to poorly differentiated thyroid carcinoma (PDTC) in terms of cellularity, nuclear atypia, and increased mitotic features with a high Ki-67 labeling index, when compared to the outer nodule (Out-N), the pathological significance of PDc remains unknown.

Defective DNA damage response (DDR) can lead to genomic instability, which is widely regarded as a key event in any carcinogenic process [2,3,4,5]. The occurrence of DNA double-strand breaks (DSBs), subsequently inducing DDR, can also be a hallmark of genomic instability [6]. TP53 binding protein-1 (53BP1) is a nuclear protein that rapidly localizes at the sites of DNA DSBs to activate the downstream repair process [7,8,9,10,11]. We previously used immunofluorescence (IF) to show that the profile of 53BP1 expression, including the number of nuclear foci which reflects endogenously occurring DDR, is altered during diverse tumorigenesis [12,13,14,15,16,17,18,19].

Several types of genetic mutations, such as those present in *BRAF, RAS, RET, TERT*-promoter (*TERT*-p)*,* and *CTNNB*, are involved in thyroid carcinogenesis [20,21,22,23]. Point mutations in *NRAS codon 61* are most common in TFTs other than follicular variants of papillary carcinoma [22,24]. *TERT*-p mutations are involved in the progression and aggressiveness of thyroid cancers and are more common in PDTC and anaplastic thyroid carcinoma than in differentiated thyroid carcinoma [23].

To elucidate the malignant potential of PDc at the molecular pathological level, this study investigated the type of 53BP1 expression, including the incidence of 53BP1 nuclear foci and *NRAS codon 61* and *TERT*-p mutations in TFTs showing NN appearance with PDc, in comparisons to the other TFTs, including AGs, FAs, minimally invasive follicular carcinomas (MFCs), and widely invasive FCs (WFCs). Our results signified genomic instability in premalignant follicles during thyroid follicular carcinogenesis and a malignant potential of abnormal type 53BP1 expression at the molecular pathological level. This study highlights the significance of PDc in NN as a precursor lesion associated with PDTC.

## 2. Materials and Methods

### 2.1. Sample Collection and Preparation

This study defined noninvasive TFT showing NN with PDc using the following parameters:

1. Well-circumscribed, well-differentiated follicular tumor macroscopically and histologically exhibits NN appearance regardless of encapsulation.
2. Inner nodule predominantly comprises PDc, such as STI-patterned follicular cells, occasionally admixed with microfollicular components.
3. Out-N comprises well-formed follicular-patterned follicular cells.
4. Tumors with metastasis or PTC-like nuclear features are excluded.

**Figure 1:**
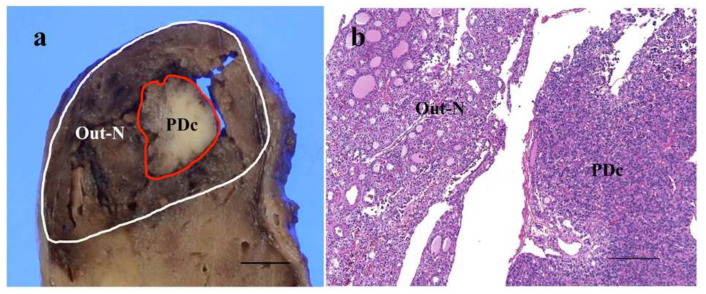
Representative image of cut-surface of thyroid follicular-patterned tumor showing nodule-in-nodule appearance with poorly differentiated component (PDc) (**a**). A histological feature of the border area between outer nodule (Out-N) and PDc at low power magnification (**b**). The scale bars indicate 5 mm in (**a**) and 200 µm in (**b**).

**Figure 2:**
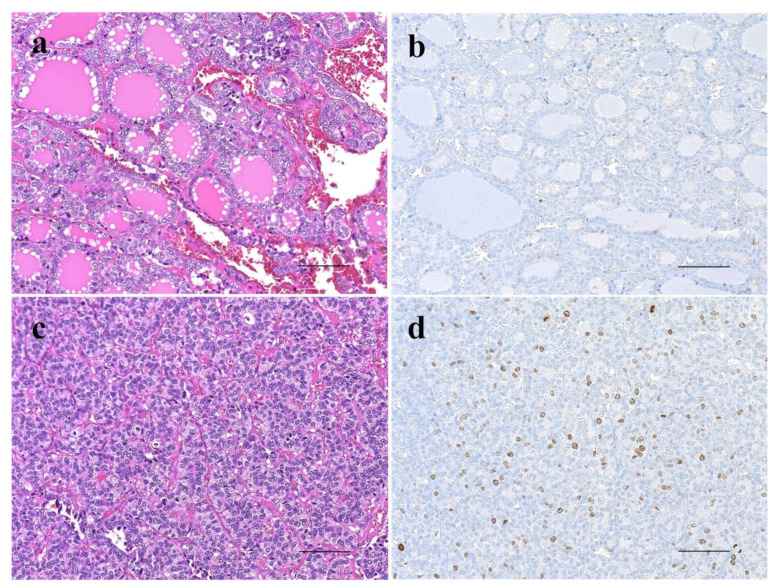
Comparison of histopathology and proliferative activity by immunohistochemistry for Ki-67 expression between outer nodule (Out-N) (**a**,**b**) and poorly differentiated component (PDc) (**c**,**d**) in thyroid follicular tumor showing nodule-in-nodule appearance. Out-N shows well-differentiated follicular pattern (**a**) and few Ki-67-positive cells (**b**), whereas PDc shows solid and nestic growth pattern (**c**) and several Ki-67-positive cells (**d**). The scale bars indicate 100 µm.

**Figure 3:**
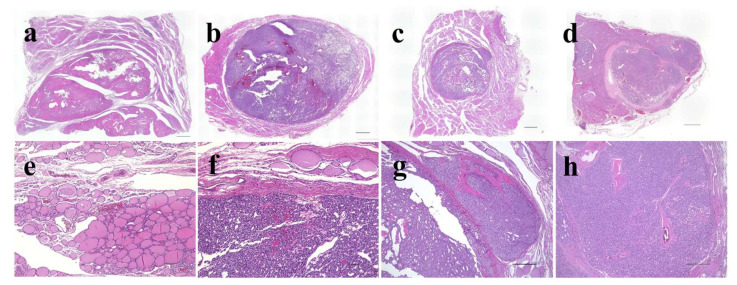
Representative histological images of thyroid follicular-patterned tumors used in this study. Adenomatous goiter (**a**,**e**); follicular adenoma (**b**,**f**); minimally invasive follicular carcinoma (**c**,**g**); widely invasive follicular carcinoma (**d**,**h**). The scale bars indicate 2 mm in (**a**–**d**), 100 µm in (**e**,**f**), and 500 µm in (**g**,**h**).

We found 16 cases (0.67%) of noninvasive TFT showing NN with PDc in a total of 2391 cases of thyroid nodules surgically resected at the Yamashita Thyroid Hospital in Fukuoka, Japan, between 2018 and 2021. These cases were available for analyses. Among 16 cases, 10 cases exhibited a solid pattern, 1 case exhibited a trabecular pattern, and 5 cases exhibited at least two STI patterns. A total of 61 cases of classical type AGs (*n* = 30) and FAs (*n* = 31) were selected as benign controls, and a total of 26 cases of MFCs (*n* = 15) including 2 cases of encapsulated angioinvasive FCs (eAFCs) with limited vascular invasion (<4 foci) and WFCs (*n* = 11) including 2 cases of eAFCs with extensive vascular invasion (≥4 foci) was selected as malignant controls from our database of total 2,391 cases of thyroid nodules. Representative pathological images of TFTs in this study are shown in Figure 1, Figure 2 and Figure 3. All available samples were formalin-fixed and paraffin-embedded (FFPE) tissues. Final diagnosis of all cases was histologically confirmed at the Department of Tumor and Diagnostic Pathology, Nagasaki University, following the diagnostic criteria of the WHO Classification of Tumors of Endocrine Organs (4th edition) [1]. The clinicopathological profiles of the patients are shown in Table 1.

### 2.2. IF Analysis for 53BP1 Expression

**Figure 4:**
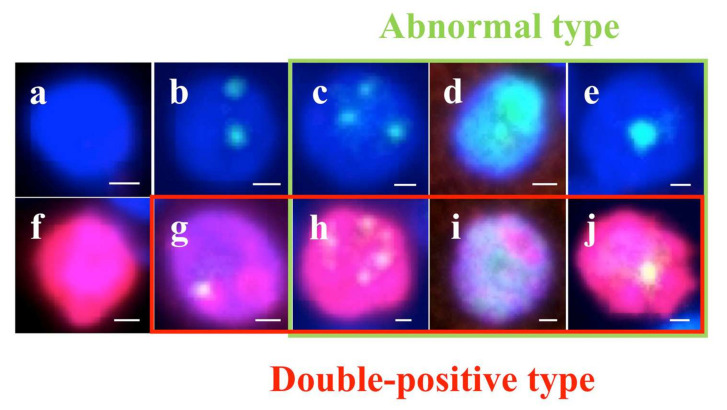
Types of TP53-binding protein 1 (53BP1) expression (green) by dual-color immunofluorescent analysis with Ki-67 expression (red). Stable type, no or faint nuclear staining (**a**,**f**); low DNA damage response (DDR) type, one or two discrete nuclear foci (NF) (**b**,**g**); high DDR type, three or more discrete NF (**c**,**h**); diffuse type, intense heterogeneous nuclear staining (**d**,**i**); and large NF type: discrete nuclear foci, which are larger than 1.0 µm (**e**,**j**). Photos surrounded by green line indicate abnormal type 53BP1 expression, and photos surrounded by red line indicate co-expression of 53BP1 and Ki-67. The scale bars indicate 2 μm.

53BP1 nuclear expression was examined by dual-color IF analysis with Ki-67 expression to assess the extent and integrity of DDR. After deparaffinization and antigen retrieval by microwave treatment in Target Retrieval Solution, pH 6.0 (Agilent Technologies, Santa Clara, CA, USA), the tissue sections of 4 µm thickness were preincubated with Dako Protein Block, Serum-Free (DakoCytomation, Glostrup, Denmark). For dual-color IF, the sections were incubated with anti-53BP1 rabbit polyclonal antibody (1:1000; A200-272A; Bethyl Labs, Montgomery, TX, USA) and anti-Ki-67 mouse antibody (1:50; MIB-1; DakoCytomation) for one hour at 20 °C. The slides were then incubated with Alexa Fluor 488-conjugated goat anti-rabbit antibody (Molecular Probes Inc., Eugene, OR, USA) and Alexa Fluor 594 F (ab’)-conjugated goat anti-mouse antibodies (Molecular Probes Inc, Eugene, OR, USA). All samples were counterstained with 4;6-diamidino-2-phenylindole dihydrochloride (DAPI-I; Vysis Inc., Downers Grove, IL, USA), analyzed, and photographed on a High Standard All-in-One Fluorescence Microscope (Biorevo BZ-X710; KEYENCE Japan, Osaka, Japan) using the Z-stack function, accumulating images of about 30 slices. All signals for 53BP1 nuclear expression were analyzed in over three viewing areas from normal and tumor parts (Out-N and PDc parts of NN), in each case at a 1000-fold magnification. 53BP1 signals were measured using the image analysis software provided with the Biorevo BZ-X710 microscope. According to our previous report [15], the type of 53BP1 immunoreactivity can be classified into five types based on the number and size of nuclear foci: (1) stable type: no nuclear staining; (2) low DDR type: one or two discrete nuclear foci; (3) high DDR type: three or more discrete nuclear foci; (4) diffuse type: intense heterogeneous nuclear staining; and (5) large nuclear foci type: discrete nuclear foci which are larger than 1.0 µm. In this study, types (3), (4), and (5) were considered abnormal types of 53BP1 expression. In addition, nuclei showing co-localization of 53BP1 nuclear foci and Ki-67 by double staining were measured. Because DDR is normally associated with cell cycle arrest, the co-expression of 53BP1 nuclear foci and Ki-67 can be considered as an indicator of impaired DDR pathway. The percentage of cells expressing each type was calculated in each case. Representative images of the type of 53BP1 expression are depicted in Figure 4.

### 2.3. DNA Extraction

To analyze *NRAS* and *TERT*-p mutations, genomic DNA (gDNA) from normal follicular and tumor areas in each case was separately extracted from FFPE tissues by macro-dissection. For cases of NNs with PDc, gDNA of Out-N and PDc areas in the tumor area were separately extracted by microdissection, with a guide slide stained with hematoxylin and eosin. Each FFPE section of 10 µm thickness was dewaxed with 80% xylene in a tube. The dewaxed sample was then washed with absolute ethanol twice and centrifuged at 15,000× *g* for 15 min at 20 °C. After drying, samples were digested with proteinase K overnight at 56 °C. DNA extraction was performed using the Maxwell RSC DNA FFPE Kit (Promega, Madison, WI, USA) and the Maxwell RSC Instrument (Promega) according to the manufacturer’s protocol. The concentration of double-stranded DNA was quantified by using a QuantiFlour ONE dsDNA system (Promega).

### 2.4. Droplet Digital PCR (ddPCR) for NRAS Codon 61 Mutation

*NRAS codon 61* mutation was analyzed by ddPCR using the *NRAS Q61* Screening Kit (catalog #12001006; BIO-RAD, Hercules, CA, USA), which detects five mutations in *NRAS codon 61* (Q61K, Q61L, Q61R, Q61H 183A > T, and Q61H 183A > C), according to the manufacturer’s protocol. A total of 20 µL of each reaction mixture containing 1.5 µL of extracted DNA was loaded into a sample well of DG8 Cartridge (catalog #1864008; BIO-RAD) followed by adding 60 µL of Droplet Generation Oil for Probes (catalog #1863005; BIO-RAD) into the oil wells. Droplets were generated by the QX200 Droplet Generator (catalog #1864002; BIO-RAD) and transferred into a clean 96-well plate. The plate was sealed with the PX1 PCR Plate Sealer (catalog #1814000; BIO-RAD), then PCR was set up in C1000 Touch Thermal Cycler (catalog #1851197; BIO-RAD). The following cycling conditions were used: (i) 95 °C for 10 min (1 cycle), (ii) 94 °C for 30 s and 55 °C for 2 min 30 s (40 cycles), (iii) 98 °C for 10 min (1 cycle), and (iv) 12 °C infinite hold. The ramp rate for cycles (i), (ii), and (iii) and for cycle (iv) was 2 °C/second and 1 °C/second, respectively. After PCR amplification, each droplet was analyzed by the QX200 Droplet Reader (catalog #1864003; BIO-RAD) and QuantaSoft^TM^ Software (catalog #1864011; BIO-RAD).

### 2.5. ddPCR for TERT-Promoter Mutation

TERT-p mutation was analyzed by ddPCR using ddPCR Supermix for Probes (catalog #1863010; BIO-RAD), primers; TERT F 5′-CAGCGCTGCCTGAAACTC-3′ and TERT R 5′- GTCCTGCCCCTTCACCTT-3, probes; and TERT mut, 5′-/56-FAM/ C+CC+C+T+TC+CGG/3IABkFQ/-3′ and TERT wt, 5′-/5HEX/C+CC+C+C+TC+CGG/3IABkFQ/-3′ (a base preceded by + is Locked Nucleic Acid). One microliter of uracil DNA glycosylase (Invitrogen, Carlsbad, CA, USA) was also added to remove uracil residues from DNA. The following cycling conditions were used: (i) 37 °C for 20 min (1 cycle), (ii) 95 °C for 10 min (1 cycle), (iii) 94 °C for 30 s and 58 °C for 2 min 30 s (40 cycles), (iv) 98 °C for 10 min (1 cycle), and (v) 12 °C infinite hold. The ramp rate for cycles (i), (ii), and (iii) and for cycle (iv) was 2 °C/s and 1 °C/s, respectively [25,26].

### 2.6. Immunohistochemical Analyses for Conventional Biomarkers of Thyroid Carcinomas

In addition to 53BP1, we analyzed the expression of p53 (monoclonal, DakoCytomation), cytokeratin 19 (CK19) (monoclonal, DakoCytomation), galectin-3 (monoclonal, Biocare Medical, Pacheco, CA, USA), and Hector Battifora mesothelial cell-1 (HBME-1) (monoclonal, DakoCytomation), which are conventional biomarkers for estimating the malignant potential of thyroid tumors, in all 16 cases of NNs and 2 cases of PDTC by immunohistochemistry using Ventana BenchMark ULTRA platform (Ventana Medical Systems Inc., Tucson, AZ, USA) according to the manufacturer’s protocol.

### 2.7. Statistical Analyses

The Jonckheere–Terpstra test was used to assess differences in the incidence of Ki-67 expression, abnormal type 53BP1 expression, and double positive-type of 53BP1 and Ki-67 expression between the histological types of TFTs. Differences in the frequency of *NRAS codon 61* and *TERT*-p mutations in TFTs were evaluated by the chi-square test. The Mann–Whitney U test was used to assess the association between the type of 53BP1 expression and the *NRAS codon 61*/*TERT*-p mutation in NNs. All statistical analyses were performed using SAS v8.2 (SAS Institute, Cary, NC, USA) and were two-tailed, with a *p*-value of < 0.05 considered statistically significant.

## 3. Results

### 3.1. Type of 53BP1 Expression in TFTs Showing NN Appearance with PDc

**Figure 5:**
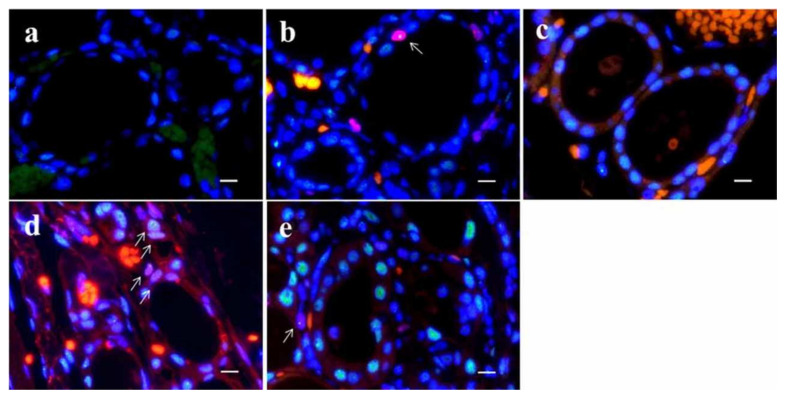
Dual-color immunofluorescence for TP53-binding protein 1 (53BP1) (green) and Ki-67 (red) in normal thyroid follicle surrounding follicular tumors. Adenomatous goiter (**a**); follicular adenoma (**b**); nodule-in-nodule appearance tumor with poorly differentiated component (**c**); minimally invasive follicular carcinoma (**d**); and widely invasive follicular carcinoma (**e**). Arrows in (**b**,**d**,**e**) indicate double-positive types. The scale bars indicate 10 µm.

**Figure 6:**
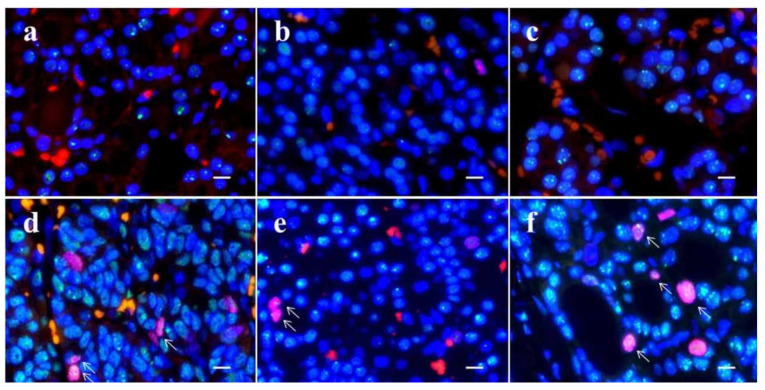
Dual-color immunofluorescence for TP53-binding protein 1 (53BP1) (green) and Ki-67 (red) in thyroid follicular-patterned tumors. Adenomatous goiter (**a**); follicular adenoma (**b**); outer nodule in nodule-in-nodule (NN) appearance tumor with poorly differentiated component (PDc) (**c**); PDc in NN (**d**); minimally invasive follicular carcinoma (**e**); and widely invasive follicular carcinoma (**f**). Arrows in (**d**,**e**,**f**) indicate double-positive types. The scale bars indicate 10 µm.

**Figure 7:**
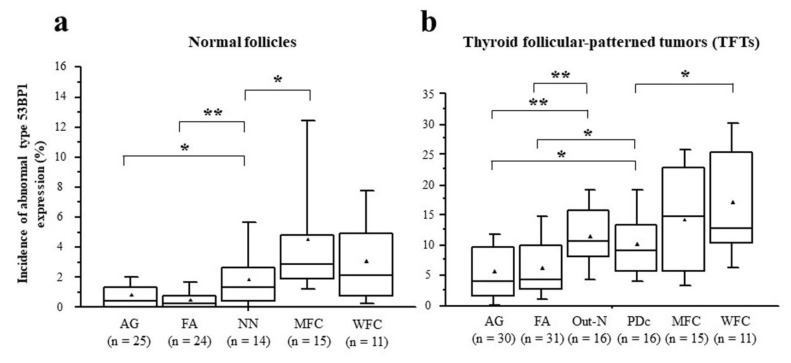
Comparison of median incidences of abnormal type 53BP1 expression in normal follicles surrounding thyroid follicular-patterned tumors (TFTs) (**a**) and in TFTs (**b**) among histological type. * *p* < 0.05, ** *p* < 0.005 by Student’s *t*-test. ▲ indicates the mean value.

Types of 53BP1 expression identified in this study are shown in Table 2, and representative images of the normal follicle surrounding TFTs and those of TFTs are depicted in Figure 5 and Figure 6, respectively. Although most normal follicular nuclei were stable or low DDR type, the frequency of appearance of abnormal type 53BP1 expression in cumulative normal follicular cells increased significantly with the type of corresponding TFTs, such as AG (0.8%), FA (0.5%), NN with PDc (1.9%), MFC (4.5%), and WFC (3.0%) (*p* < 0.0001, Jonckheere–Terpstra test). Normal follicles surrounding NN with PDc and FC showed a significantly higher frequency of abnormal type 53BP1 expression when compared with normal follicles surrounding benign nodules, such as AG and FA (Figure 7a). Analysis of double staining with Ki-67 expression revealed that co-localization with 53BP1 nuclear expression was restricted to normal follicles surrounding FC, with co-localization rates of 0.40% for MFC and 0.28% for WFC but very low co-localization rates for benign nodules (Table 2). The co-localization of 53BP1 nuclear expression with Ki-67 in normal follicles was significantly associated with the histological type of TFTs (*p* = 0.0189).

The incidence of abnormal type 53BP1 expression in cumulative nuclei increased significantly in tumor areas from benign to malignant nodules, such as AG (5.6%), FA (6.5%), Out-N (11.7%), PDc (10.3%), MFC (14.2%), and WFC (17.1%) (*p* < 0.0001, Jonckheere–Terpstra test). When compared with benign nodules, both Out-N and PDc in NN had a significantly higher frequency of abnormal types but were lower than WFC (Figure 7b). Furthermore, double staining with Ki-67 expression demonstrated that the frequency of double-positive cells with 53BP1 nuclear expression in cumulative nuclei increased significantly from benign to malignant nodules, such as AG (0.03%), FA (0.12%), Out-N (0.08%), PDc (0.36%), MFC (0.67%), WFC (0.68%) (*p* < 0.0001, Jonckheere–Terpstra test). The statistical analysis revealed that the frequency of double-positive cells in PDc was significantly higher than in AG (*p* < 0.001), FA (*p* = 0.0124), and Out-N (*p* = 0.0167) but not in MFC (*p* = 0.1050) and WFC (*p* = 0.716).

### 3.2. Frequency of NRAS Codon 61 and TERT Promoter Mutations and Their Association with Abnormal Type 53BP1 Expression in TFTs

Frequencies of *NRAS codon 61* and *TERT*-p mutations in TFTs detected by ddPCR are shown in Table 3. There were no *NRAS codon 61* and *TERT*-p mutations detected in normal follicles surrounding TFTs. The *NRAS codon 61* mutation was the most frequently detected mutation in both Out-N and PDc tumor areas, accounting for 56.3% of all cases. Statistical analysis revealed that the frequency of *NRAS codon 61* mutation in both Out-N and PDc was significantly higher than in AG (3.3%, *p* < 0.001) and FA (20.0%, *p* = 0.0125), but not in MFC (26.7%, *p* = 0.0953) and WFC (36.4%, *p* = 0.3096). *TERT*-p mutation was the most frequently detected mutation in WFC (36.4%), followed by PDc of NN (25.0%), MFC (13.3%), and Out-N of NN (12.5%).

The association between abnormal type 53BP1 expression and *NRAS codon 61*/*TERT-p* mutations in NN with PDc is shown in Table 4. No correlation was observed between the *NRAS codon 61* and the frequency of abnormal type 53BP1 expression or co-expression of 53BP1 and Ki-67. The frequency of co-expression of 53BP1 and Ki-67 in the *TERT-p* mutant (0.73%) was higher than that in wild type (0.24%); however, a significant difference was not observed (*p* = 0.1016).

### 3.3. Immunohistochemical Analyses for Conventional Biomarkers of Thyroid Carcinomas in NN with PDc and PDTC

The immunohistochemical data obtained in each case are summarized in Supplemental Table S1, along with the clinicopathological features, molecular analyses, and the type of 53BP1 expression. Representative images for immunohistochemical results in PDc of NN and PDTC are shown in Supplementary Figures S1 and S2, respectively. Among the test subjects, case no. 2 was not analyzed for immunohistochemistry because of a shortage of available additional sections. Among the other 15 cases of NNs, 9 cases (60%) of PDc were positive for two or three markers, while 3 cases (20%) of Out-N were positive for two or three markers. Both cases of PDTC were positive for all three markers. Furthermore, the level of p53 immunoreactivity, including a mild p53 positivity suggesting wild type, were higher in PDc (93.3%) than in Out-N (40%). One case (case no. 7) of PDc showed a block-positive p53 immunoreactivity along with two cases of PDTC, signifying a mutant type. This case was positive for three conventional markers. In contrast with the immunoreactivity observed for conventional markers, little or no expression of 53BP1 was observed in PDTC. No association between immunohistochemical results and type of 53BP1 expression was apparent in our cases.

## 4. Discussion

This study demonstrated a stepwise increase in abnormal type 53BP1 expression in the tumorigenesis of TFTs in the order of AG < FA < MFC < WFC. We previously reported that the number of 53BP1 nuclear foci increases with the malignant potential of TFTs, such as FA, MFC, and WFC [16]. Our recent study using liquid-based cytology samples obtained from resected TFTs suggested that the frequency of abnormal type 53BP1 expression in TFTs could be an attractive candidate biomarker for distinguishing FC from FA [27]. This study demonstrated that the frequency of abnormal type 53BP1 expression in NN with PDc was significantly higher than in benign nodules, but not in FCs, and that the incidence of *NRAS codon 61* and *TERT*-p mutations in NN with PDc was comparable to that of FCs. Immunohistochemistry for conventional biomarkers to estimate the malignant potential of thyroid nodules also revealed a higher level of immunoreactivity in PDc than in Out-N of NN (Supplementary Figure S1). Furthermore, we found a higher level of co-expression of 53BP1 and Ki-67 in PDc when compared with Out-N of NN and benign nodules. Because the DDR process is normally conducted in non-cycling cells, co-localization of 53BP1 and Ki-67 expressions can be considered as a sign of impaired DDR machinery, which is a key event in carcinogenesis [14]. FCs most frequently exhibited co-expression of 53BP1 and Ki-67 among TFTs. Thus, we suggest that NN with PDc may be a true neoplastic lesion, and PDc especially seems to be a component of neoplasia possessing malignant characteristics, such as defective DDR with a high proliferative capability, which is a phenotype of genomic instability during follicular carcinogenesis. Interestingly, in contrast to PDc in NN, 53BP1 immunoreactivity was obviously decreased in PDTC cases (Supplementary Table S1). Our previous study also revealed a loss of 53BP1 immunoreactivity in invasive fronts of advanced esophageal cancers [17]. We speculate that cells at the tumor front that exhibit an invasive capability lack the ability to respond to DNA damage, indicating a possibility of increased genomic instability and more aggressive phenotypes. The present observations in PDTC suggest that once the thyroid cancer reaches a poorly differentiated state exhibiting a mutant p53 and *TERT*-p mutation, the ability to respond to DDR is lost, leading to a decreased 53BP1 expression. 

The current study also revealed abnormal type 53BP1 expression with a significant increase in Ki-67 expression in normal follicles surrounding FC. Along similar lines, our previous studies revealed an increased number of nuclear 53BP1 foci in non-neoplastic epidermis at sun-exposed sites [13] and in normal urothelium surrounding bladder carcinoma [15]. These cases also exhibited higher Ki-67 labelling indices, suggesting replication stress, even though the cells appeared to be normal. Normal follicles surrounding FC may also exhibit abnormal type 53BP1 expression representing minor genotoxic injuries, such as aging-related reactive oxygen species (ROS), which may be associated with thyroid carcinogenesis [28].

Our previous experiments based on IF analysis of FFPE tissues showed that the presence of 53BP1 NF was increased in irradiated rat thyroid glands in a dose-dependent manner and that the presence of 53BP1 and γH2AX nuclear foci was frequently co-localized in human TFTs as well as irradiated rat thyroid glands, suggesting endogenous activation of the DDR pathways in tumor cells as a hallmark of genomic instability [12]. So far, we have found that abnormal type 53BP1 expression is closely associated with carcinogenesis in several organs [12,13,14,15,16,17,18,19]; for instance, both large nuclear foci and diffuse patterns are significantly associated with high-grade urothelial carcinoma with chromosomal instability and poor prognosis in bladder cancers [15]. In addition, abnormal types of 53BP1 expression are associated with chromosomal instability, a parameter of prognosis in gastric MALT lymphoma [19]. Thus, we believe that analyzing 53BP1 expression by IF can be useful for estimating the level of genomic instability and malignant potential of human tumors.

Noninvasive TFT showing NN appearance with PDc is a rare type of thyroid nodule, which reportedly has indolent behavior [29]. Despite a short follow-up period of 27 (7–43) months, no recurrences or metastases were observed in our cases. Rivera et al. examined eight cases of noninvasive encapsulated thyroid tumors of follicular cell origin with high-grade poorly differentiated features and found no recurrence after a median follow-up of 11.9 years and *NRAS codon 61* mutations to be the most frequent oncogenic event [29]. However, Kobayashi et al. suggested that FC could be transformed from a benign thyroid tumor and show an NN appearance on ultrasonography [30]. One case of an encapsulated, noninvasive, and follicular thyroid neoplasia with high-grade poorly differentiated features with multiple metastases was reported [31]. Our analysis indicated a heterogeneity in distribution of nuclei expressing both 53BP1 and Ki-67 immunoreactivity, as well as a Ki-67 labeling index in NN with PDc, suggesting a difference in the extent of defective DDR machinery between Out-N and PDc in NN despite identical *NRAS codon 61* mutation status in both components. Therefore, we speculate that PDc develops from well-differentiated Out-N harboring *NRAS* mutations, which can enhance proliferative activity during carcinogenesis [32,33], eventually progressing to malignant potential by impaired DDR machinery and *TERT*-p mutation during follicular carcinogenesis. Some authors have also suggested the role of *NRAS exon 61* mutation in the progression of PDTC from well-differentiated thyroid carcinoma [32,34] and *TERT*-p mutations as the most common alterations in PDTC [23,35]. Thus, considering its histological similarity with PDTC, PDc in NN might be a precursor lesion of PDTC.

There are a few limitations of this study. First, the sample size is small as noninvasive TFTs showing NN with PDc is rare. Second, this study is a single-center study. Thus, it is necessary to increase the number of cases in a multicenter study to examine the long-term course of PDc in NN.

## 5. Conclusions

This study demonstrated that normal follicles surrounding FCs showed a high level of abnormal type 53BP1 expression, suggesting genomic instability in premalignant follicles during thyroid follicular carcinogenesis. Furthermore, the level of abnormal type 53BP1 expression and the incidence of *NRAS codon 61* and *TERT*-p mutations in PDc were comparable to FCs, suggesting a malignant potential at the molecular pathological level. Because co-localization of 53BP1 NF and Ki-67 expression is an indicator of an impaired DDR pathway, the development of PDc in NN may be associated with DDR impairments after harboring an *NRAS* and *TERT*-p mutations. Thus, we should pay more attention to PDc in NN as a precursor lesion associated with PDTC.

## Figures and Tables

**Table 1:** Clinicopathological profiles of the patients in this study.

Histological Type	*n*	Age (Range)	Men/ Women	Size [mm] (Range)	Nodal Metastasis	Distant Metastasis
AG	30	54.8 (27–79)	8/22	45.5 (15–87)	N/A	N/A
FA	31	51.1 (20–80)	6/25	38.1 (13–86)	N/A	N/A
NN with PDc	16	55.5 (36–79)	5/11	42.0 (11–80)	N/A	N/A
MFC	15	52.9 (22–78)	4/11	30.8 (7–58)	0 (0%)	0 (0%)
WFC	11	57.5 (31–84)	3/8	47.6 (19–144)	0 (0%)	2 * (18.2%)

AG, adenomatous goiter; FA, follicular adenoma; NN, nodule-in-nodule appearance tumor; PDc, poorly differentiated component; MFC, minimally invasive follicular carcinoma; WFC, widely invasive follicular carcinoma; N/A, not applicable. * 2 WFCs had distant metastasis in lungs and bones.

**Table 2:** Types of TP53-binding protein 1 (53BP1) in thyroid follicular-patterned tumors (TFTs) identified by immunofluorescence.

	Histological Type	*n*	Counted Nuclei	Ki-67 Expression (%)	Type of 53BP1 Expression (%)			Co-Expression of 53BP1 and Ki-67 (%)
					Stable	Low DDR	Abnormal	
Normal follicles	AG	25	6242	0.1 ± 0.2	91.4 ± 6.3	7.8 ± 5.5	0.8 ± 1.1	0.01 ± 0.06
	FA	24	6730	0.3 ± 0.5	94.4 ± 3.0	5.1 ± 2.8	0.5 ± 0.7	0.02 ± 0.08
	NN with PDc	14	3084	0.2 ± 0.4	90.9 ± 5.0	7.2 ± 4.2	1.9 ± 1.9	0.00 ± 0.00
	MFC	15	5049	1.3 ± 2.8	86.3 ± 6.4	9.2 ± 3.5	4.5 ± 4.2	0.40 ± 1.22
	WFC	11	3258	0.9 ± 1.2	88.4 ± 6.3	8.7 ± 4.7	3.0 ± 2.9	0.28 ± 0.64
				*p* = 0.0056 *			*p* < 0.0001 *	*p* = 0.0189 *
TFTs	AG	30	16,518	0.1 ± 0.2	74.6 ± 12.3	19.8 ± 8.9	5.6 ± 5.0	0.03 ± 0.06
	FA	31	21,568	0.5 ± 0.6	74.8 ± 10.4	18.6 ± 6.3	6.5 ± 4.8	0.12 ± 0.23
	NN with PDc							
	Out-N	16	5111	0.6 ± 0.6	73.5 ± 15.4	14.8 ± 11.7	11.7 ± 5.5	0.08 ± 0.21
	PDc	16	7680	2.5 ± 2.5	77.7 ± 14.9	12.0 ± 11.6	10.3 ± 5.5	0.36 ± 0.40
	MFC	15	16,790	2.1 ± 1.7	68.1 ± 17.4	17.7 ± 10.7	14.2 ± 8.7	0.67 ± 0.62
	WFC	11	12,782	2.3 ± 1.6	61.6 ± 19.0	21.3 ± 11.7	17.1 ± 9.2	0.68 ± 0.48
				*p* < 0.0001 *			*p* < 0.0001 *	*p* < 0.0001 *

DDR, DNA damage response; AG, adenomatous goiter; FA, follicular adenoma; NN, nodule-in-nodule; PDc, poorly differentiated component; MFC, minimally invasive follicular carcinoma; WFC, widely invasive follicular carcinoma; Out-N, outer nodule. * Jonckheere–Terpstra test.

**Table 3:** *NRAS codon 61* and *TERT*-promoter mutations in thyroid follicular-patterned tumors detected by droplet digital PCR.

Histological Type	*n*	*NRAS codon 61* Mutation (%)	*TERT*-Promoter Mutation (%)
AG	30	1 (3.3)	N/A
FA	30	6 (20.0)	N/A
NN with PDc			
Out-N	16	9 (56.3)	2 (12.5)
PDc	16	9 (56.3)	4 (25.0)
MFC	15	4 (26.7)	2 (13.3)
WFC	11	4 (36.4)	4 (36.4)

AG, adenomatous goiter; FA, follicular adenoma; NN, nodule-in-nodule appearance tumor; Out-N, outer nodule; PDc, poorly differentiated component; MFC, minimally invasive follicular carcinoma; WFC, widely invasive follicular carcinoma; N/A, not applicable.

**Table 4:** Association between type of TP53-binding protein 1 (53BP1) expression and *NRAS codon 61* and *TERT-promoter* mutations in nodule-in-nodule appearance tumor with poorly differentiated component.

Type of *NRAS codon 61/TERT*-Promoter	*n*	Abnormal Type 53BP1 Expression (%)	Co-Expression of 53BP1 and Ki-67 (%)
*NRAS codon 61*			
Wild	7	9.8 ± 4.5	0.54 ± 0.41
Mutant	9	10.6 ± 6.4	0.22 ± 0.36
*TERT*-promoter			
Wild	12	9.5 ± 5.9	0.24 ± 0.29
Mutant	4	12.7 ± 3.9	0.73 ± 0.53

## Data Availability

All data generated in the study are available in the manuscript and its supplement. Raw data used in this study are not available due to patient confidentiality.

## Supplementary Materials

The following supporting information can be downloaded at: https://www.mdpi.com/article/10.3390/cancers14153577/s1, Figure S1: Representative immunohistochemical images (a: weak and focal nuclear p53, b: focal CK19, c: diffuse galectin-3, d: diffuse HBME-1) of poorly differentiated component (PDc) in nodule-in-nodule (NN) appearance thyroid tumor. The scale bars indicate 100 µm. Figure S2: Representative immunohistochemical images (a: diffuse nuclear p53, b: diffuse CK19, c: diffuse galectin-3, d: focal HBME-1) of poorly differentiated thyroid carcinoma. The scale bars indicate 100 µm. Table S1: Summary of clinicopathological data of nodule-in-nodule appearance tumor with poorly differentiated component and poorly differentiated thyroid carcinoma.
