# Supplementary material for: Molecular Pathological Characteristics of Thyroid Follicular-Patterned Tumors Showing Nodule-in-Nodule Appearance with Poorly Differentiated Component
Source: Cancers (Basel). 2022 Jul 22;14(15):3577. doi: 10.3390/cancers14153577 (PMC9331311; doi:10.3390/cancers14153577)
Supplement: Supplementary file 1 [file cancers-14-03577-s001.zip › Supplemental_figures.pdf]

Supplemental FigureS1

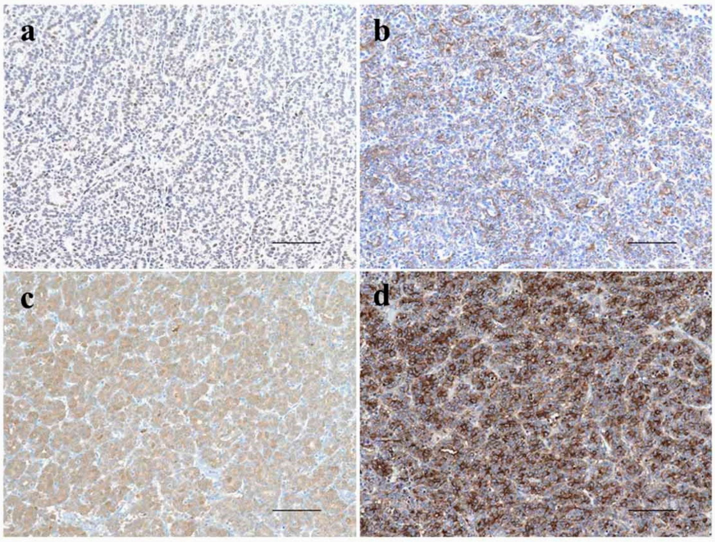

Figure S1: Representative immunohistochemical images (a: weak and focal nuclear p53, b: focal CK19, c: diffuse galectin-3, d: diffuse HBME-1) of poorly differentiated component (PDc) in nodule-in-nodule (NN) appearance thyroid tumor. The scale bars indicate 100 μm.

Supplemental FigureS2

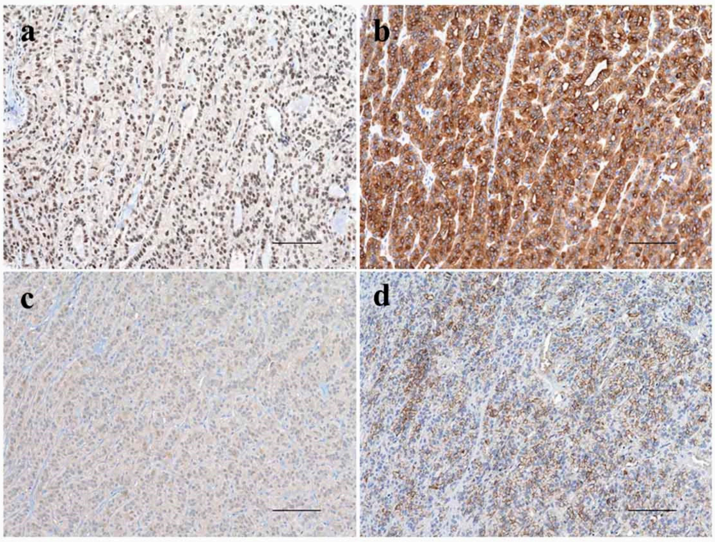

Figure S2: Representative immunohistochemical images (a: diffuse nuclear p53, b: diffuse CK19, c: diffuse galectin-3, d: focal HBME-1) of poorly differentiated thyroid carcinoma. The scale bars indicate 100 μm.
