# Supplementary material for: Molecular Pathological Characteristics of Thyroid Follicular-Patterned Tumors Showing Nodule-in-Nodule Appearance with Poorly Differentiated Component
Source: Cancers (Basel). 2022 Jul 22;14(15):3577. doi: 10.3390/cancers14153577 (PMC9331311; doi:10.3390/cancers14153577)
Supplement: Supplementary file 1 [file cancers-14-03577-s001.zip › Supplemental_table.pdf]

Supplemental Table S1. Summary of clinicopathological data of nodule-in-nodule appearance tumor with poorly differentiated component and poorly differentiated thyroid carcinoma.

	No.	Age	Sex	Size (mm)		STI pattern	Necrosis	Mitoses (/10HPF)	*NRAS codon61*		*TERT promoter*		p53		CK19		Galectin-3		HBME-1		53BP1 abnormal type (%)		Co-expression of 53BP1 and Ki-67 (%)	
				Out-N	PDc				Out-N	PDc	Out-N	PDc	Out-N	PDc	Out-N	PDc	Out-N	PDc	Out-N	PDc	Out-N	PDc	Out-N	PDc
NN with PDc	1	40	F	16	7	S	-	3	WT	WT	WT	WT	-	±	-	±	-	-	-	+	12.9	12.3	0.00	0.13
	2	79	M	41	19	S	-	0	Mut	Mut	WT	WT	N/E	N/E	N/E	N/E	N/E	N/E	N/E	N/E	18.0	19.4	0.00	0.00
	3	76	F	44	11	T>S	-	5	Mut	Mut	WT	C228T	-	±	-	-	-	-	+	±	21.9	10.9	0.00	0.00
	4	47	F	16	10	S	-	0	Mut	Mut	WT	WT	-	-	±	±	-	+	±	+	4.1	4.1	0.00	0.34
	5	63	F	68	22	S	+	5	WT	WT	C228T	C228T	±	±	±	±	-	±	-	+	14.4	18.5	0.00	1.16
	6	58	M	11	3	S	-	3	WT	WT	WT	WT	-	±	-	-	-	±	-	+	8.0	7.8	0.00	0.39
	7	47	F	55	17	S>T	-	5	Mut	Mut	WT	WT	-	+	±	±	-	+	-	+	8.6	3.4	0.00	0.00
	8	49	F	40	8	S	-	4	Mut	Mut	WT	WT	±	±	-	-	-	-	-	+	9.7	5.1	0.00	0.00
	9	50	F	24	11	S	-	3	Mut	Mut	WT	WT	±	±	-	±	-	±	±	±	5.6	7.5	0.00	0.00
	10	63	M	80	9	T>I	-	19	WT	WT	C228T	C228T	-	±	±	±	-	-	±	±	10.1	10.8	0.25	0.67
	11	69	F	50	11	S	-	0	WT	WT	WT	WT	±	±	-	-	-	-	-	-	14.0	5.2	0.00	0.74
	12	60	F	43	18	S>I	-	4	WT	WT	WT	WT	-	±	-	-	-	±	-	-	3.0	6.3	0.00	0.00
	13	64	F	31	7	S	-	0	WT	WT	WT	WT	±	±	-	-	-	-	-	-	17.5	8.1	0.85	0.74
	14	42	M	18	9	S	-	0	Mut	Mut	WT	WT	-	±	±	+	±	-	-	+	11.0	14.3	0.13	0.11
	15	45	F	63	8	T	-	4	Mut	Mut	WT	WT	-	±	±	-	±	-	+	+	8.2	20.6	0.00	0.65
	16	36	M	72	32	T>I	-	3	Mut	Mut	WT	C228T	±	±	-	+	-	-	-	+	19.2	10.5	0.00	1.03
PDTC	1	75	F	17		S>T	+	3	WT		C250T		+		+		+		±		0.6		0.00	
	2	69	F	94		S>T	-	3	WT		C228T		+		+		+		±		0.0		0.00	

NN, nodule-in-nodule appearance tumor; PDc, poorly differentiated component; PDTC, poorly differentiated thyroid carcinoma; Out-N, outer nodule; S, solid; T, trabecular; I, insular; CK19, cytokeratin 19; HBME-1, Hector Battifora mesothelial cell-1; 53BP1, TP53-binding protein 1; F, female; M, male; WT, wild type; Mut, mutant type; N/E, not examined because of shortage of available sections; -, negative; ±, focal-positive; +, diffuse-positive
